# Preventing unintentional home injuries among children: exploring the perceptions of Iranian health professionals

**DOI:** 10.1017/S1463423619000835

**Published:** 2019-10-30

**Authors:** Atena Barat, Michael Craig Watson, Caroline A. Mulvaney

**Affiliations:** School of Medicine, University of Nottingham, Nottingham, UK

**Keywords:** childhood unintentional injury, health professional, home safety, injury prevention, Iran, qualitative

## Abstract

**Background:**

Health professionals are key stakeholders who potentially have important roles in preventing unintentional child home injuries. This study aimed to identify facilitators and barriers to the prevention of unintentional child home injuries perceived by health professionals.

**Design:**

A generic qualitative study involving semi-structured interviews.

**Setting:**

The capital city of Iran.

**Method:**

Data for this study were collected through 28 in-depth interviews with health professionals. Purposive sampling was conducted from three areas of Tehran based on their socio-economic development.

**Findings:**

Thematic data analysis yielded nine overarching themes: prioritising child home injury, knowledge, the nature of injury and injury prevention, child-related factors, parent-related factors, living environment, cultural issues, resources and management.

**Conclusions:**

Health professionals can potentially be supportive to meet families’ needs. However, further support and resources will be required if they are to fully develop their potential in preventing injuries in the home. The lack of a national action plan was a significant constraint for health professionals.

## Introduction

Child unintentional injuries are a global public health problem and are a major cause of mortality and disability in the under-fives, with the highest burden in low- and middle-income countries (Peden *et al.*, 2008). In Iran in 2005, a total of 2879 disability-adjusted life years per 100,000 were due to unintentional injuries in children aged under five years (Naghavi *et al.*, 2010). The Ministry of Health & Medical Education (MOHME) in Iran reported that 5.5% of the population suffering injuries were children aged 0–4 years old, ranked as the fifth highest risk group for injuries in terms of age in 2010 (Ghadiri-Afshar and Hadadi, 2010). However, this report included all types of injuries, that is, both intentional and unintentional, in all age groups in all settings.

Health professionals (HPs) are stakeholders whose role in terms of preventing injuries is affected by injury prevention policies (Freeman, 1984; Mack *et al.*, 2016). HPs work in different sectors of a health system and can undertake a variety of activities for injury prevention. As they have frequent contacts with parents and their children, it provides them with many opportunities for prevention work (DiGuiseppi and Roberts, 2000; Watson and Errington, 2016). They can also be involved in local safety groups, lobbying and campaigning on safety issues, leadership and advocacy roles, and changing legislation (Sibert, 1991; Kendrick, 1994; DiGuiseppi *et al.*, 1996).

Several studies have confirmed that HPs’ work can facilitate a reduction of home hazards, enhance safety equipment usage and in some cases decrease injury rates (Bass *et al.*, 1993; Towner *et al.*, 1993; King *et al.*, 2005; Kendrick *et al.*, 2013). Despite their positive attitude towards injury prevention, they have raised concern over their abilities and communication skills (Morrongiello *et al.*, 1995; Soori and Motlagh, 1999; Woods, 2006; Watson *et al.*, 2007).

Understanding the perspective of HPs of injury prevention is important because of their supportive roles for families. However, there is a dearth of literature, particularly in Iran, related to the attitudes of HPs towards the prevention of unintentional child injuries at home. In order to bridge this knowledge gap, the current study therefore aimed to explore HPs’ perspectives regarding barriers and facilitators that affect them in the implementation of interventions targeting prevention of unintentional child home injury. Such findings will inform the development of supportive programmes to promote home safety for young children.

## Methods

### Study design

This qualitative study followed an interpretivist epistemological approach, to evoke data about experiences and views of HPs regarding child injuries (Locke *et al.*, 2010). A generic qualitative approach was adopted in this research as its highly inductive nature is useful for addressing practical problems (Kahlke, 2014).

### Study sampling

This study was conducted in Tehran, the capital of Iran. Purposeful sampling of information-rich cases was used to cover all regions of Tehran and achieve diversity in the samples. In doing so, the city was classified into three areas based on their socio-economic development level: poor, middle and affluent (Mohamadzadeh-Asl *et al.*, 2010). One health centre inside each area was randomly selected as a place of recruitment.

Inclusion criteria for participants were:

- working at the selected health centres or relevant organisations
- having relevant work experience or potentiality to be involved in programmes related to the prevention of child injury.

There were two types of HPs: direct and indirect. The former have immediate contact with the public and provide health services for them in health centres, for example, practitioners in Family Health Unit, whereas the latter work in health networks or universities with responsibility to support direct HPs in performing health initiatives, for example, head of Health Network.

The researcher visited study sites and through discussions with the managers and by using an organisational chart, identified and was introduced to potential participants who were available the day of the visit. The researcher met possible interviewees, passed them an invitation letter enclosing an information sheet and discussed the purpose of the study. They had 1 week to consider participation and to ask any questions about the study and to declare their agreement to participate in the research. Those who agreed to take part in the study were contacted to make an appointment for an interview. This process was continued until data saturation was achieved.

The recruitment and interviews took approximately four months, from January to April 2014. Thirty-one professionals were approached; but after further explanation of the study three declined to take part due to time constraints.

### Data collection

This study gathered in-depth information through semi-structured interviews with open questions conducted in the workplace at the interviewees’ convenience.

An interview guide designed to address the research objectives was designed from a review of the literature by the lead author (A.B.) and validated by two other authors (M.W. and C.M.) (see Table 1). A pilot interview was conducted to enhance the study rigor through testing the efficacy of the research instrument and to elicit potential unanticipated difficulties in a practical research environment (Welman and Kruger, 2001). The pilot data were included in the main analysis because the research instrument did not need major revision (Teijlingen and Hundley, 2001).

**Table 1.** Individual interview guide

No.	Interview questions
1	What are the families’ expectations of you as a health professional for the prevention of child home injuries?
2	What are the needs of families for controlling the problem, according to your evaluation?
3	How do you estimate the cooperation of families for controlling this problem?
4	What are your needs for prevention of child home injuries in a satisfied way? How can you be supported?
5	Have you received any training for preventing child home injuries? Prompt: – If yes what are they?
6	From your point of view, what can be done better to improve the prevention of child home injuries in your catchment area?
7	Is there any disciplined and structured programme in the health system for prevention of child home injuries?Prompt: – If yes, please name
8	What hinders you to prevent child home injuries at your local area?

Interviews were performed by the lead investigator (A.B.) in Persian and audio-recorded with permission. Interviews ranged from 16 to 54 min in duration, with a mean time of 32 min depending on the extent of information that an interviewee shared.

### Data analysis

All audio recordings were transcribed verbatim, then analysed using inductive thematic analysis (Braun and Clarke, 2006). The first author (A.B.) read the transcripts to identify blocks of text that implicitly or explicitly addressed research objectives. These blocks of text were given labels (codes), and by reading through transcripts several times, new codes emerged from the data set or text was assigned to the existing codes. Similar codes were gathered into more conceptual categories and develop themes to address research questions. This iterative coding process continued until no further new codes appeared and all concepts were developed to support the themes and sub-themes. To ensure that respondents’ attitudes were truly represented through the first author’s interpretations (A.B.), 25% of the study transcripts were double-coded independently by two other authors (M.W. and C.M.), this is considered good practice (Miles and Huberman, 1984).

This study was cross-language research as data were collected and analysed in Persian while findings were presented in English. Translation into English was limited to selected quotes, codes and themes. Back translation with the help of a bilingual translator was used to check the accuracy of translation.

Involving more than one person in the analysis and providing audit trail enhanced the study rigour and its transparency, which enables readers to more easily assess the study and to compare it with others.

Each participant was labelled with a code, to preserve anonymity (Dahlgren *et al.*, 2007), consisting of two letters and a number. The former refers to groups that a participant belongs to (direct or indirect/socio-economic region); and the latter indicates the order of conducting an interview (e.g., DL3 refers to the third interview with a direct HP working in a low socio-economic region).

Analyses were performed by hand using Microsoft Office Word 2013 software.

This research was approved by The University of Nottingham, Faculty of Medicine and Health Sciences Research Ethics Committee (OVSA11072013SNMP) and the MOHME in Iran. Further details of the research method are presented elsewhere (Barat *et al.*, 2017).

## Findings

The characteristics of the 28 HPs who participated in the interviews are described in Table 2. They were working in three levels of the health system: university, health network and health care centre. Just over half of the participants (*N* = 15) were managers. The main role of six interviewees was health education, and eight were in charge of recording injury incidents.

**Table 2.** Health professionals’ characteristics

		Number of health professionals (*n* = 28)	
Characteristics		Indirect (*N* = 18)	Direct (*N* = 10)
Organisation	University Vice-Chancellor for Health	7	0
	Health Network	8	0
	Health Centre	3	10
Role	Management	11	4
	Health education	2	4
	Data recording	5	2
Years of experience	Less than 5	0	1
	6–10	2	2
	11–15	3	2
	16–20	9	5
	More than 20	4	0
Level of education	Diploma	0	2
	Undergraduate degree (BSc/MD)	12	8
	Post-graduate degree (MSc/MPH/PhD)	6	0
Gender	Female	15	10
	Male	3	0

Respondents had a wide range of working life from 4 to 27 years. Those with the most experience were generally indirect HPs. All participants except those in health liaison were educated in health-related disciplines such as medicine, midwifery, nursing and public health.

Barriers and facilitators for tackling child home injuries were identified through HPs’ perspectives. Analysis of interview data yielded nine major themes (Table 3).

**Table 3.** The perception of health professionals about facilitators and barriers

Themes	Sub-themes	
Prioritising child home injury		
Knowledge		
The nature of injury and injury prevention		
Child-related factors		
Parent-related factors	Socio-economic status	
	Mothers’ employment	
	Parents’ age	
	Parents’ practice	
Living environment		
Cultural issues		
Resources	Financial	
	Physical	
	Human	
	Data	
	Rules	
	Time	
Management	Planning	
	Organising	Lack of leading organisation
		Current health care system
		Collaboration and coordination
	Evaluation	

### Prioritising child home injury

The majority of respondents highlighted that the general public and authorities have negative attitudes towards giving priority to the subject and prefer focusing on disease and treatment issues. Additionally, nearly a quarter of participants (who were mostly indirect HPs) stated that the problem and the implementation of primary prevention initiatives is beyond the scope of MOHME’s interventions as well as their own scope of work:

We have lots of other problems and do not have time to get involved in issues which are not in our work schedules…

(DL9)

### Knowledge

Nearly half of the respondents believed that families cannot properly play their protective role for children because they are unaware of injury risk factors, the outcomes of child home injuries and their rights to ask for services in relation to the issue. Some HPs stated that they themselves do not have enough knowledge regarding this subject as their university training did not cover it.

In contrast, two indirect HPs mentioned two facilitators regarding the issue. Firstly, the general public have basic literacy to understand and apply the educational materials, and secondly, experts and MOHME policymakers have required knowledge for tackling the problem:

If we want to do an ideal intervention, we know its rationale and reasonable mechanisms. We also have access to national and international evidences. Moreover, we have gained experiences during our recent trial and error activities.

(IH17)

### The nature of injury and injury prevention

Some participants determined the inherent features of injury as a barrier. They suggested that injury incidents are probable, uncertain and unpleasant for families to think about in advance and adopt precautions. They also believed that injury is a cross-cutting problem that cannot be resolved through merely MOHME’s interventions.

According to their perspectives, the small numbers of mortalities due to home injuries compared to the death rate from road traffic injuries (RTIs) lead decision makers to particularly concentrate on RTI and ignore home injuries. Moreover, one interviewee stated that home injury as a public health concept is a new idea, and many people, including policymakers, are unaware of it. According to the point of view of two participants, the prevention of home injuries required intrusion into the domestic sphere by the state to an intolerable degree according to Iranian custom.

### Child-related factors

Based on the perspective of two interviewees, the intrinsic characteristics of under-fives are barriers to the prevention of child home injuries, as expressed by the participant:

… children at this age do not obey instructions, that is, when mothers tell their children not to touch a dangerous object, they act conversely.

(IM7)

### Parent-related factors

#### Socio-economic status

A few interviewees stated that high socio-economic status (SES) families are more highly educated and less accepting of the information provided by health centres. In contrast, one HP considered low SES of families as a facilitator, as these family groups show more enthusiasm for education, believe in the expertise of HPs and take their advice seriously. However, some interviewees mentioned that being in a low economic status can impede preventive measures because meeting the required safety standards in home is costly and they cannot afford it. Similarly, some respondents considered that being in high SES families had advantages:

In high SES families, mothers have higher educational degrees and enough information regarding health subjects. Even if we do not intervene for a problem they do it themselves. When we have an advice or a programme, their SES allows them to take them seriously and do self-study about the issue.

(IH16)

#### Mothers’ employment

A few respondents indicated that mothers’ employment is a barrier because the responsibility of their children’s supervision is delegated to others. They also explained that employed mothers encountered a lack of time to use educational services provided by health centres.

Additionally, one of the participants stated that the ‘stay at home’ status of mothers can be an enabler for preventing injury as such mothers are present with their children more and able to protect them from possible hazards.

#### Parents’ age

Some respondents cited that younger parents have a lack of experience and knowledge:

Some mothers are too young and do not have enough experience, for example, electrocution due to putting a nail into outlet shows that parents are unfamiliar with some injury risk factors.

(DM1)

However, one HP viewed the young age of parents as a facilitator:

Since mothers are young and the child is their first or second one, they are interested in the issue. They welcome and have high motivation to follow the training.

(DL3)

#### Parents’ practice

A few respondents considered parents’ practice as a facilitator which protects children, for example, putting hazardous items out of the reach of children. Nevertheless, a few participants stated that some mothers are careless and not attentive to their children’s needs, as the following interviewee described:

A child climbed a shoe storage cabinet and fell, which led to his death. This was completely due to his parents’ negligence, because we have covered these items in our educational classes.

(IL9)

In respect of families’ practice in relation to the health system, a few interviewees indicated that people had weak cooperation regarding the implementation of previous health initiatives. Conversely, some participants identified the good cooperation of parents with the health system regarding current and future health initiatives as a facilitator for the prevention of child home injuries:

Families are usually welcome to our education, especially if the subject is tangible, evidence-based and scientific. They are receptive to our advice and usually apply them because they want to protect their children.

(DL7)

### Living environment

A quarter of interviewees believed that the quality of living environments in terms of size and structure increases the probability of injury incident. They stated that small home size brings injury hazards within the reach of children and some home structures are unsafe due to using non-standard poor-quality construction materials.

### Cultural issues

A few interviewees believed that some aspects of the prevailing societal culture negatively influence families’ performance and encourage them to ignore safety precautions. A participant noted:

Due to our culture people rush for walking in children, I can say that 100% of parents buy baby walkers. It is an item among babies’ accessories. We advise mothers not to use them …

(DL3)

Moreover, they discussed that the predominant culture of both high and low SES areas enhances mothers’ deprivation of safety education opportunities because the former exerts a negative attitude towards public sector services and the latter does not accept women’s participation in social activities outside home.

### Resources

#### Financial resources

Most of the participants stated that the lack of a budget was a barrier to controlling child home injuries, and initiatives such as education and advertising are costly. As one participant remarked:

The injury programme is oppressed in the health system. I can certainly confirm that in the last two years we have not received any budget for injury programme.

(IL10)

#### Physical resources

Some interviewees stated that the lack of facilities and educational materials impedes them from preventing injuries:

One of our barriers in the health centre is lack of basic facilities for holding a good educational programme. If we had at least a TV and a fixed video, we could transmit our educational films.

(DM9)

On the contrary, some respondents indicated that access to facilities such as software for data collection and educational leaflets enabled their work:

Injury recording software has great potentialities and capabilities, although it needs some revision. If it is filled out, it can give us detailed information regarding all involved factors with rural and urban division.

(IL10)

#### Human resources

Most of the participants emphasised that the health system suffers from the shortage of human resources, particularly trained experts for injury prevention. Some informants concluded that some components of initiatives were ignored and the quality of work decreased due to the multiplicity of roles of staff and a large number of customers.

#### Data

The majority of participants indicated that there are no accurate data regarding child home injuries due to the lack of a surveillance system. Some participants stressed that there are injury data in the country, but it is unreliable and over-generalised. They attributed poor quality of data to deficiencies in data collection, for example, offline data capture at hospitals and the lack of enforcement for data collection.

#### Rules

Some indirect HPs believed that safety interventions for reducing injuries require law enforcement for the home setting. As one participant voiced:

Our administrative barrier is lack of law, for example, the child of a janitor drowned in a home pool and the landlord did not accept to install fence around the pool. We do not have legal permission to enter a private property for safety checking, or including safety tips as prerequisites of having home pools.

(IH15)

#### Time

Almost all participants explained that the quality of their communication with families is affected by lack of time. A participant discussed this issue:

Families are in a hurry. They come to get services and go… Health centres are only opened in the mornings and there is always a lack of time. I focus on important issues and mothers leave me with lots of questions in their minds.

(DM1)

### Management

A few participants ascribed barriers or facilitators for tackling injuries to the quality of performing three duties of a manager: planning, organising and evaluation.

#### Planning

More than half of the participants indicated that there is no goal, guidance or national programme on injury issues from headquarters:

In fact, no goal is defined by authorities for solving the problem. They have not planned for it. All the relevant initiatives such as education and data collection are temporary, without considering community’s needs.

(IH18)

A few respondents believed that current programmes such as Well-Child Care (WCC) could not help in controlling child injuries as their approaches are different from injury prevention. Conversely, most of the interviewees believed that some of the ongoing initiatives were facilitators for reducing child home injuries, citing vaccination, WCC, Safe Community and particularly education as current opportunities that can be used for tackling the issue.

#### Organising

- Lack of leading organisation

A few respondents mentioned that lack of a leading organisation for child home injury was an obstacle to dealing with the problem. Parallel or missing activities and lack of coordination between departments and organisations were mentioned as undesirable outcomes.

- Current health care system

The majority of interviewees stated that the current health system is itself a barrier to solving the problem in terms of its function and structure. They explained that centralised planning in headquarters brings about difficulties such as a one-way top-down relationship and a lack of autonomy for local interventions. Moreover, they criticised the weak coverage of health network in urban areas as well as inconsistencies in the organisational structure of upper and lower levels of the health system.

- Collaboration and coordination

Most of the interviewees identified weak collaboration and communication between two main departments of MOHME (Department of Health and Department of Treatment) and their subsets. Furthermore, a few participants explained that inconsistency and lack of interaction between different organisations impede success in reducing child home injuries. A participant from MOHME voiced:

If I produce a 15-minute educational programme regarding children’s safety and ask the national media to transmit it, I should pay 150m Tomans [£30,000], whereas both of us [MOHME and national media] use national income. This is a part of current inconsistencies.

(IH17)

In contrast, a few participants indicated that inter-sector collaboration between hospitals and health networks for data collection as well as inter-organisational collaboration between the health system and other organisations for educational programmes are the facilitators.

#### Evaluation

A few participants believed that programmes have not been evaluated, which they considered to be a barrier for controlling child home injuries:

Since we do not have any feedback regarding both our programmes and the rate of child home injuries, we do not know whether we are successful or not.

(DL9)

## Discussion

This study sought to understand how HPs perceive and experience barriers and facilitators for the prevention of unintentional home injuries among young children through interviews with 28 informants from different sections of Iran health system. Findings of this study corroborate with findings from previous research (Woods, 2006; Smithson *et al.*, 2011) and theories such as social ecological model (Gielen and Sleet, 2003) that in injury prevention, several factors are influencing the practice of HPs.

More than half of the interviewed HPs stated that Iran has no national action plan exclusively addressing child home injuries, which may be the result of the lack of a leading organisation for the issue. Despite this perception, the review of general health policies affirms that MOHME was explicitly appointed in 2014 as the leading organisation in health-related issues such as injuries (Akbari, 2014). Since our data collection was conducted in late 2013 and early 2014, the interviewees may have been unaware about the new policy and MOHME needed more time to show the results of its responsibility.

The lack of a national action plan appeared to be a significant constraint for HPs, as it has negative effects on other areas. According to the majority of HPs, child injury has been proposed as a subject by MOHME for health networks and centres to include in their educational programmes. Nevertheless, since there is no exclusive national action plan for the issue, no resources are allocated to it, which means that health centres must use resources of other initiatives to cover the issue if they decide to do so. Therefore, child injury is easily deprioritised due to a number of reasons including lack of knowledge about the issue among staff and the cultural perception that it is a parental responsibility of the private domestic sphere.

In the current study, the absence of training was highlighted as a barrier; this is mentioned in studies from other countries (Woods, 2006; Watson *et al.*, 2007). Similarly, the number of duties in their role was perceived as another challenge as injury prevention was placed ancillary to other responsibilities. These results support previous studies (Cohen and Runyan, 1999; Lupton *et al.*, 2000; Kendrick *et al.*, 2003; Mack *et al.*, 2016). Moreover, findings from international studies are compatible with our results regarding inadequate funding, time constraints, poor teamwork, lack of confidence, personal experience, and lack of a single source of information and training materials as other influential factors (Cohen and Runyan, 1999; Gielen *et al.*, 1997; Lupton *et al.*, 2000; Peltzer, 2001; Woods, 2006; Mack *et al.*, 2016). The role of policymakers in supporting this group in terms of training as well as legislative and engineering measures has been emphasised by other authors (Woods, 2006).

## Strengths and limitations of the study

This study provides first-hand insights into HPs’ views regarding what influences their practices for preventing child injuries at home. The range of participants and its highly inductive nature were beneficial in providing a rich description of the topic, adding depth to the existing knowledge, and helping gain new holistic insights in an Iranian context.

Nevertheless, the findings must be considered in light of some methodological considerations. Given that the study approach is interpretivism rather than a positivism one, subjectivity is justifiable and inevitable (Benton and Craib, 2011); but, the study adhered to the standard criteria of qualitative study such as double coding and peer-debriefing to ensure its trustworthiness. In addition, the knowledge produced has limited generalisability because the data were collected from a relatively small population in a particular locale. However, diversity among participants and detailed description of the study context and methodology will allow readers to make transferability inferences (Shenton, 2004).

## Implications

Although HPs had positive attitudes for tackling this important public health problem, they believed that there were serious deficiencies in terms of resources (e.g., financial, human and time). However, they thought that this could be remedied by having a national action plan for the prevention of child home injuries.

World Health Organization (Peden *et al.*, 2008) emphasised the need for an evidence-based action plan to prevent child injuries which can strengthen national and local commitment and action. This action plan should be integrated with the national child health strategy and localised according to the prevailing societal culture. Training programmes, social and financial support of injured children and their families should be included in the plan. The report also highlighted that prior to the onset of the plan, the accountability for the issue should be explicitly clarified and a relevant focal person within the health ministry designated (Peden *et al.*, 2008). France and Sweden are good leads to follow, whose national action plans make injury control a major goal of their society (Peden *et al.*, 2008).

Therefore, having a national multi-sector safety promotion programme would make child injury a national priority and an issue for concern and debate at both national and local levels. It would allow the development of cost-effective interventions that have been used successfully in other countries providing that they are adapted to the new social context (Peden *et al.*, 2008; Watson and Errington, 2016).

Our findings suggest that in order to support HPs implementing injury prevention initiatives, including educational ones, careful consideration needs to be given to safety legislation and regulation throughout the country. It is important to note that the ‘three E’s’ approach, Education, Enforcement, Environment modification, has been recommended by WHO and other leading experts in the field (Gielen and Sleet, 2003; Christoffel and Gallagher, 2006; Peden *et al.*, 2008; Watson and Errington, 2016).

Furthermore, this study argues that HPs must be well prepared to effectively fulfil their duties regarding injury prevention. Therefore, it is suggested that the subject of injury prevention is integrated into the curricula of health-related professions particularly medicine, midwifery, nursing and public health. The provision of ongoing education can also keep HPs up to date with best practice and latest developments, for example, free access to TEACH-VIP E-Learning training curriculum (WHO, 2016). It is also important that MOHME develop capacity-building publications and training resources for HPs, according to national society needs, values and injury status.

## Conclusions

This study highlights a multitude of barriers and facilitators, which hinder or enable HPs to prevent child injuries in Iran. HPs practices can primarily be supported by introducing a national action plan for the prevention of unintentional child injuries at homes. The findings inform a significant policy debate that may contribute to the development of context-specific solutions for child injuries. There is considerable potential for young children in Iran to have greater protection from unintentional injuries and live more safely in their homes.
